# Seed culture pre-adaptation of *Bacillus coagulans* MA-13 improves lactic acid production in simultaneous saccharification and fermentation

**DOI:** 10.1186/s13068-019-1382-2

**Published:** 2019-02-28

**Authors:** Martina Aulitto, Salvatore Fusco, David Benjamin Nickel, Simonetta Bartolucci, Patrizia Contursi, Carl Johan Franzén

**Affiliations:** 1 Department of Biology, University of Naples Federico II, 80126 Naples, Italy; 2 Division of Industrial Biotechnology, Department of Biology and Biological Engineering, Chalmers University of Technology, 412 96 Gothenburg, Sweden

**Keywords:** *Bacillus coagulans*, Simultaneous saccharification and fermentation, Pre-adaptation, Wheat straw, Hydrolysate

## Abstract

**Background:**

Lignocellulosic biomass is an abundant and sustainable feedstock, which represents a promising raw material for the production of lactic acid via microbial fermentation. However, toxic compounds that affect microbial growth and metabolism are released from the biomass upon thermochemical pre-treatment. So far, susceptibility of bacterial strains to biomass-derived inhibitors still represents a major barrier to lactic acid production from lignocellulose. Detoxification of the pre-treated lignocellulosic material by water washing is commonly performed to alleviate growth inhibition of the production microorganism and achieve higher production rates.

**Results:**

In this study, we assessed the feasibility of replacing the washing step with integrated cellular adaptation during pre-culture of *Bacillus coagulans* MA-13 prior to simultaneous saccharification and lactic acid fermentation of steam exploded wheat straw. Using a seed culture pre-exposed to 30% hydrolysate led to 50% shorter process time, 50% higher average volumetric and 115% higher average specific productivity than when using cells from a hydrolysate-free seed culture.

**Conclusions:**

Pre-exposure of *B. coagulans* MA-13 to hydrolysate supports adaptation to the actual production medium. This strategy leads to lower process water requirements and combines cost-effective seed cultivation with physiological pre-adaptation of the production strain, resulting in reduced lactic acid production costs.

**Electronic supplementary material:**

The online version of this article (10.1186/s13068-019-1382-2) contains supplementary material, which is available to authorized users.

## Background

Lactic acid (LA) is a widely used platform chemical with applications in food, cosmetic, pharmaceutical, and chemical industries [1]. For instance, LA is an important building block for the production of poly-lactic acid (PLA), a bioplastic [2]. LA can be produced via microbial fermentation or chemical synthesis. However, fermentation provides significant advantages, such as a reduced environmental impact and a lower raw material cost [1]. Nonetheless, one of the major costs in fermentative LA production is still represented by the pure sugar solutions (e.g., glucose, sucrose, and lactose) used as carbon sources [3, 4]. Indeed, the preparation of these substrates from agricultural feedstocks is expensive, given the purification processes required [5]. On the other hand, sustainable sugar-containing materials (e.g., such as syrups, juices and molasses) can be used for a more cost-effective production of LA [4]. For example, several starch-degrading *Lactobacillus* species are used for one-step LA fermentation of starch-containing materials [6]. However, this may inconveniently compete with the supply of foods and feeds [7].

To overcome this drawback, lignocellulosic biomass is an abundant and sustainable feedstock [8, 9], and thus a promising alternative source of raw material for LA production via microbial fermentation. The utilization of lignocellulose requires several process steps, including a thermochemical pre-treatment under harsh conditions of high temperature and low pH [10–12] to enhance the accessibility of enzymes to the biomass during the subsequent saccharification step [13], in which the polysaccharides are cleaved into fermentable sugars. The use of agricultural residues to produce such sugar solutions also requires substantial purification, both before and after production of the chemical [5]. Saccharification can be performed separately from fermentation (separate hydrolysis and fermentation, SHF) or combined as simultaneous saccharification and fermentation (SSF) [14]. An advantage of SSF is the reduced end-product inhibition experienced by the hydrolytic enzymes due to direct sugar consumption by the microbial fermentation. On the other hand, given the operational temperature and pH of the hydrolytic enzymes typically used for the saccharification (50–55 °C and pH 5.0–5.5), optimal fermentation performance can be achieved if thermophilic microorganisms/enzymes are used [15–17]. After pre-treatment and saccharification, the fermentability of the biomass is generally hampered by toxic compounds, such as furfural, 5-hydroxymethyl furfural (HMF), and soluble phenolics, which are released from the biomass during the pre-treatment [18]. These chemicals represent a major barrier in the development of production processes from lignocellulosic biomass [19], because they affect the fermentation rate by inhibiting microbial growth. One strategy to overcome inhibition is to detoxify lignocellulosic materials by washing the solid residue with water. However, the cost of this additional step is higher than its benefits [19, 20]; therefore, alternative strategies to alleviate inhibition need to be investigated.

A promising strategy to decrease microbial inhibition is pre-adaptation, in which the fermenting microorganism is exposed to biomass-derived inhibitors during seed cultivation (cell propagation step). Thereby, the microorganism adapts to these inhibitors and shows an improved fermentation performance in the subsequent SSF, which is reflected in shorter lag phase as well as higher growth rate and yield [21, 22]. Nonetheless, a crucial requirement to use this approach is an intrinsic inducible tolerance of the microorganism towards biomass-derived inhibitors, which allows its propagation in the presence of such toxic compounds. For instance, the *Bacillus coagulans* strain DSM2314 showed improved fermentation performance when it was pre-cultivated in a medium supplemented with a non-lethal amount of furfural. In particular, the authors observed a significant cell elongation upon exposure to furfural that was linked to the upregulation of genes involved in the synthesis of the cell walls. Interestingly, such a morphological change is a typical stress response in bacilli and it is related to a diminished vulnerability to cell autolysis [23].

Recently, a new strain of *B. coagulans*, named MA-13, was isolated from canned beans manufacturing residues and found to secrete soluble cellulolytic enzymes into the culture supernatant. MA-13 has temperature (55 °C) and pH (5.5) optima for cell growth that are comparable with those required by the fungal hydrolytic enzymes used for the biomass saccharification [24]. Moreover, MA-13 tolerated the toxicity of biomass-derived growth inhibitors well when cultivated on sucrose (molasses) in the presence of high concentrations of pre-treatment hydrolysate (up to 95%) [24]. These features make MA-13 an attractive biocatalyst for the conversion of lignocellulosic residues into valuable chemicals. However, the robustness of MA-13 and its ability to produce LA from solid lignocellulosic raw materials in SSF configuration remains to be investigated.

In this study MA-13 was used as a microbial biocatalyst to produce LA from steam exploded wheat straw in simultaneous saccharification and fermentation. We show that the presence of pre-treatment hydrolysate in the seed culture medium results in pre-adaptation of the strain to the biomass-derived inhibitors and higher rates of LA production than for the non-adapted control.

## Results and discussion

### Overall scheme of the pre-adaptation strategy and SSF

**Fig. 1:**
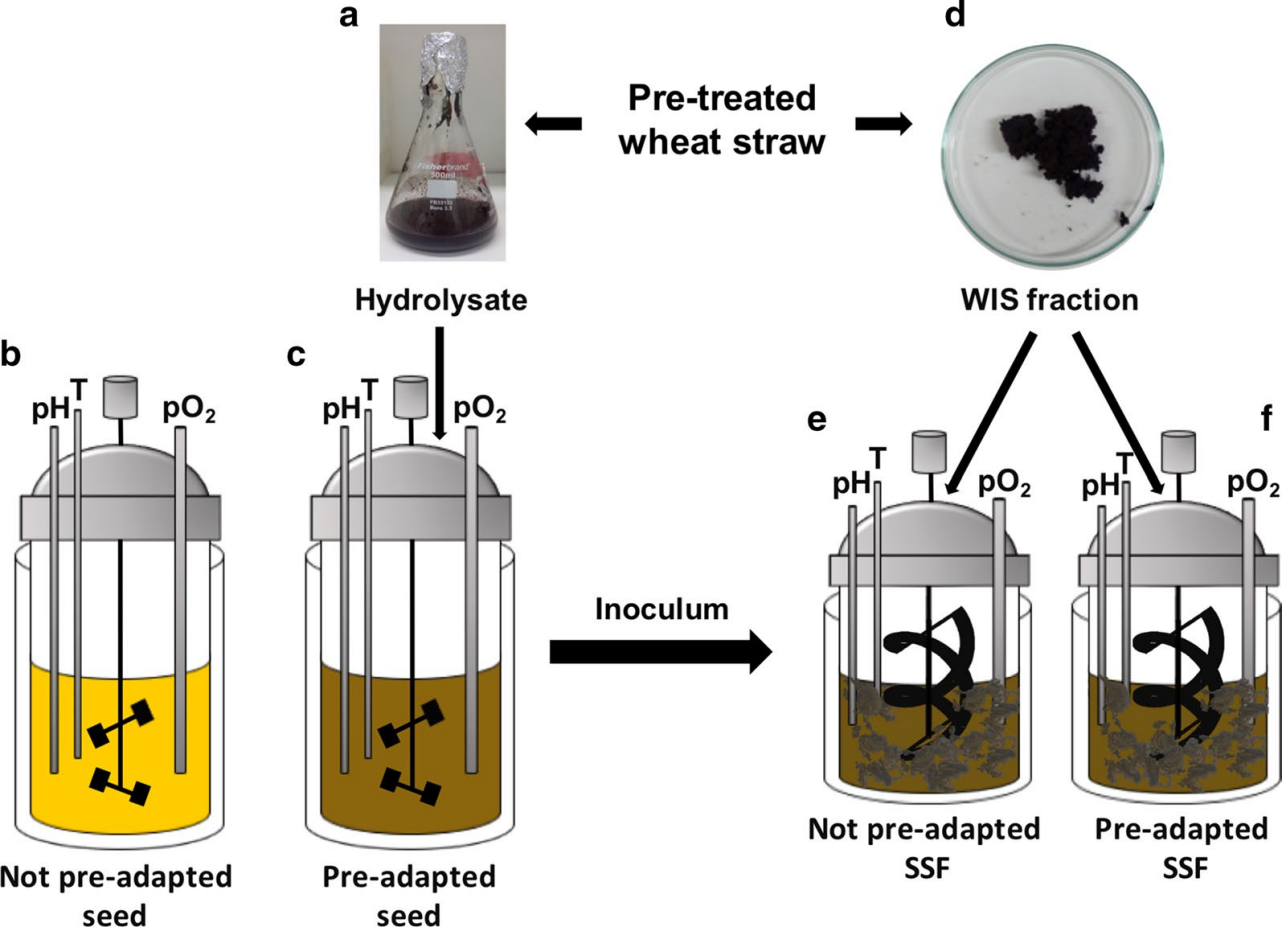
Schematic illustration of the pre-adaptation strategy and SSF. Wheat straw was pre-treated by acid-catalysed steam explosion and the biomass slurry was separated by filtration into a liquid fraction (**a**) and a water-insoluble solids (WIS) fraction (**d**). Unadapted (**b**) and pre-adapted (**c**) seed cultures were inoculated in the SSF bioreactor (**e**, **f**), respectively. Whereas only the liquid fraction (hydrolysate) was used for seed cultivations, both hydrolysate and WIS (brown fibres in **e** and **f**) were added to the SSF

The underlying hypothesis of this work is that the fermentation performance, in terms of lactate yields on consumed glucose (glu) and lactate productivity, depends on the physiological state of *B. coagulans* MA-13 after seed cultivation. To assess our hypothesis, we tested whether MA-13 seed cultures could be adapted to the inhibitors present in the hydrolysate (Fig. 1a). To do so, anaerobic seed cultures were grown in hydrolysate-free medium (Fig. 1b) as well as in media supplemented with different amounts of hydrolysate (Fig. 1c). The seed cultures were inoculated to SSF bioreactors (Fig. 1e, f) containing the solid fraction of the wheat straw biomass (Fig. 1d) mixed with a hydrolytic enzyme cocktail. To evaluate the fermentation performance of the different seed cultures, cell growth as well as lactic acid productivities and yields were determined.

### Effect of the hydrolysate supplementation on the seed cultures

**Fig. 2:**
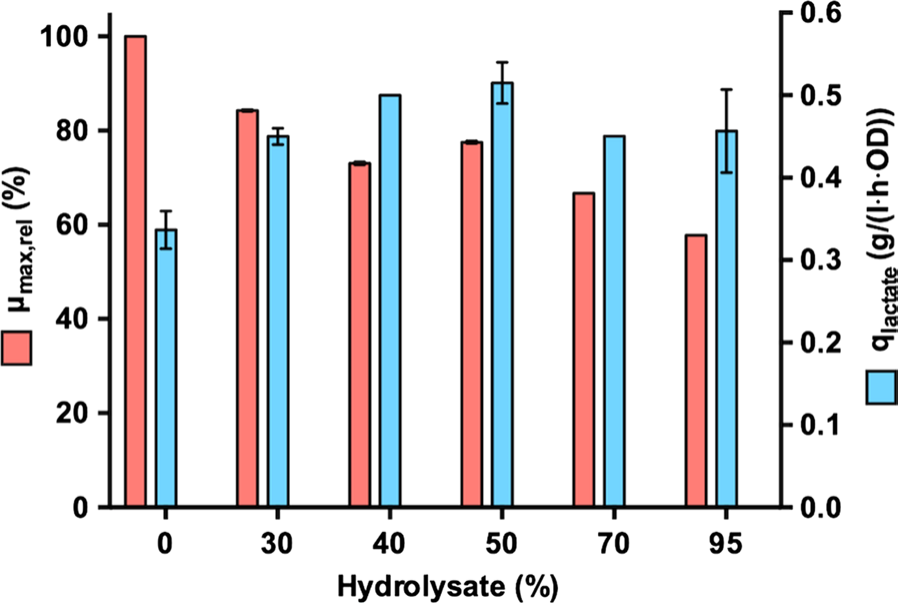
Seed cultures behavior upon supplementation of pre-treatment hydrolysate. Seeds were cultivated anaerobically in 3.6 L bioreactor vessels at 55 °C and pH 5.5 in 1 L working volume of molasses-based medium. Relative maximum specific growth rate (*μ*_max,rel_(%), red bars) and maximum specific lactate production rate (*q*_lactate_ (g/(L h OD)), blue bars) are reported for seeds propagated in hydrolysate-free medium (0) as well as in media prepared with 30%, 40%, 50%, 70% and 95% hydrolysate. Relative maximum specific growth rate is reported as percentage of the maximum specific growth rate (h^−1^) in hydrolysate-free medium (Partially adapted from Figure 8 and Table 2 in [24])

The addition of 95% (v/v) hydrolysate in the seed media reduced the maximum specific growth rate (*μ*_max_) of MA-13 by 42% compared to hydrolysate-free medium (Fig. 2). However, the maximum specific productivity of LA in the seed culture increased from 0.34 g/(L h OD) in the hydrolysate-free medium to 0.51 g/(L h OD) at 50% hydrolysate (Fig. 2). Higher concentrations, instead, resulted in a reduction of the maximum specific LA productivity. The stimulated LA productivities in hydrolysate-containing media may be explained by higher cellular energy requirements caused by stress response mechanisms. Under anaerobic conditions cells can overproduce energy in terms of adenosine triphosphate (ATP) and reducing power only through an enhanced sugar fermentation that results in increased LA production. On the other hand, if the stress due to inhibitors becomes too high, the LA productivity instead declines, as illustrated by the media containing more than 50% pre-treatment hydrolysate (Fig. 2).

These observations are promising in the light of what has been reported in the literature so far [20, 25–29]. In fact, other *B. coagulans* strains used for the production of LA in SSF have been cultivated in hydrolysate-free rich media, likely because of their susceptibility to the biomass-derived growth inhibitors (Table 1). The use of hydrolysate-containing medium provides an advantage over the conventional seed cultivation, because it allows reducing the amount of clean water used for microbial propagation. Another important aspect affecting the economy of the whole LA production process is the cost of the carbon source used during cell propagation. In this regard, unlike other studies [20, 25–29], the use of molasses (i.e., a renewable, easily available and relatively inexpensive source of sugars) as well as the presence of hexoses in the hydrolysate make the whole process more economically feasible.

**Table 1:** Overview of recent studies on LA production in SSF using different *B. coagulans* strains

Strain	Biomass			Process						Lactic acid		References
	Raw material	Pre-treatment	Detoxification	Seed culture medium	Fermentation set-up	Temperature (°C)	pH	Enzyme load	Process time (h)	LA avg. vol. productivity (g/(L h))	Yield (g/g)	
MA-13	Wheat straw	H_2_SO_4_-steam explosion	–	SM-0%	SSF	55	5.5	10 FPU/g_WIS_	30	1.11	1.23^a^/1.24^b^/0.27^c^	This work
				SM-30%					15	1.74	1.02^a^/1.10^b^/0.27^c^	
DSM 2314	Birch wood	Steam explosion	Water washing	TSNT	SSF	50	5.5	2 mg/g_DM_	120	0.35	0.79^b^	[28]
CC17	Bagasse	Sulfite pulping	Water washing	GCY	Fed-batch SSF	50	5.0–5.5	10 FPU_cellulase_/g_cellulose_ 120 U_xylanase_/g_hemicellulose_	120	0.92	0.72^b^/0.60^d^	[25]
LA204	Corn cob	NaOH	Water washing	YEX	Fed-batch SSF	50	6.0	30 (FPU/g_cellulose_)	90	1.37	0.77^c^	[29]
		NH_3_-H_2_O_2_							90	1.32	0.74^c^	
			–						72	1.10	0.43^c^	
LA204	Corn stover	NaOH	Water washing	GY or XY	Fed-batch SSF	50	6.0	30 (FPU/g_stover_)	60	1.63	0.68^c^	[26]
IPE22	Wheat straw	H_2_SO_4_-steam explosion	–	mMRS	SSCF	50	5.0	20 FPU/g_cellulose_	60	0.43	0.46^c^	[27]
DSM 2314	Wheat straw	Lime	–	GY	Fed-batch SSF	50	6.0	n.r.	55	0.68	0.43^c^/0.81^d^	[20]

*DM* dry matter, *FPU* filter paper unit, *n.r.* not reported, *U* unit; *WIS* water-insoluble solid

*SM-0%*; *SM-30%* Seed medium containing 0% and 30% hydrolysate, respectively (see composition in the paragraph “Strain and media”. )

*mMRS* modified MRS: 10 g/L peptone; 10 g/L beef extract; 5 g/L yeast extract; 2 g/L K_2_HPO_4_; 0.2 g/L MgSO_4__7H_2_O; 0.05 g/L MnSO_4__4H_2_O; 10 g/L glucose

*GY* 10 g/L glucose; 10 g/L yeast extract; 2 g/L (NH_4_)_2_HPO_4_; 3.5 g/L (NH_4_)_2_SO_4_; 10 g/L Bis–Tris; 0.02 g/L MgCl_2_·6H_2_0 and 0.1 g/L CaCl_2_·2H_2_0

*TSNT* 10 g/L tryptone; 5 g/L soytone; 3 g/L NaCl and 1 g/L Tween 80

*GCY* 20 g/L glucose; 2.5 g/L corn steep powder; 1 g/L yeast extract; 1 g/L NH_4_Cl; 0.2 g/L MgSO_4_ and 10 g/L CaCO_3_

*GY (or XY)* 50 g/L glucose (or xylose) and 10 g/L yeast extract

*YEX* 10 g/L xylose and 10 g/L yeast extract

^a^g of lactic acid/g of consumed glucose

^b^g of lactic acid/g of glucose released from cellulose

^c^g of lactic acid/g of total dry matter

^d^g of lactic acid/g of total sugars released from the biomass

### Lactic acid production by pre-adapted seed cultures in SSF

After filtration, the solid residue used for the production of LA in SSF retains a certain content of hydrolysate. Therefore, it still contains soluble growth inhibitors that can interfere with the microbial metabolism during SSF. So far, several strains of *B. coagulans* have been used for the production of LA from lignocellulosic biomasses in SSF configuration (Table 1). In most of these cases, detoxification of the solid residue by washing with water was performed prior to SSF, to remove the growth inhibitors from the solid residue. An example is the strain LA204, which was used for SSF with both washed and unwashed corncob [29]. This strain showed higher LA average volumetric productivity (1.32 g/(L h)) and yield (0.74 g/g) when the biomass was subjected to water washing detoxification than when no washing was done (1.10 g/(L h) and 0.43 g/g), (see Table 1). However, this approach is not industrially sustainable because of the extra vessels, equipment and clean water that would be required for the detoxification.

In this study we have instead tested if pre-exposure of the seed culture to the biomass-derived inhibitors could alleviate their detrimental effects during the subsequent SSF, and thereby eliminate the need for washing the solid residue.

### SSF using a (cells/water insoluble solids) ratio of 0.01 g_cells_/g_WIS_

**Fig. 3:**
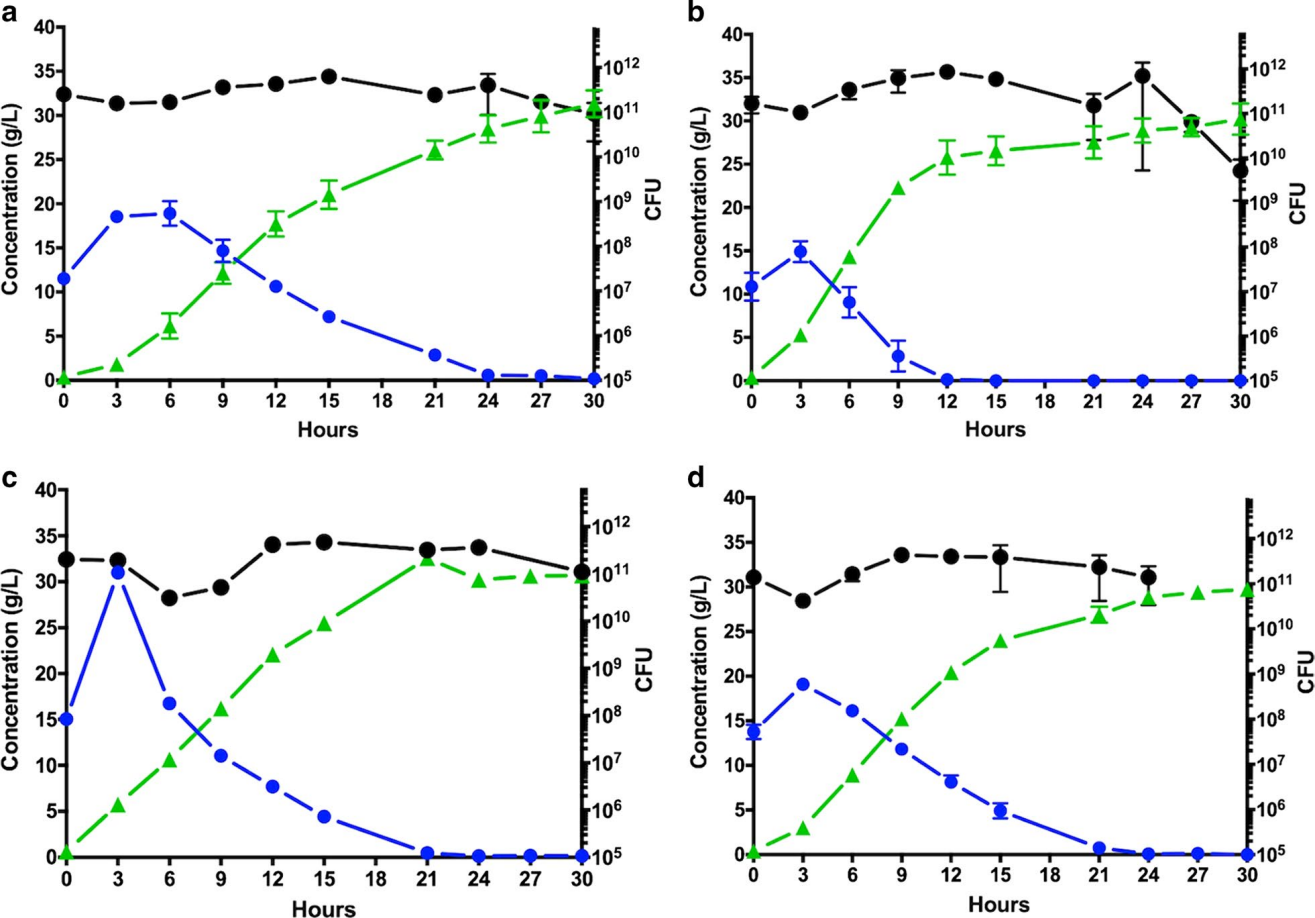
Time-course of batch SSF performed with *B. coagulans* MA-13 at a cell/biomass ration of 0.01 g_cells_/g_WIS_. SSF experiments were carried out anaerobically in 3.6 L BioEtOH double-jacket flat-bottom vessels at 55 °C and pH 5.5. Pre-hydrolysis of the WIS fraction was carried out for 30 min by adding 10 FPU/g_WIS_ of Cellic CTec 2 before inoculating cells from the seed cultures. The fermentation medium (10% WIS from pre-treated wheat-straw) was inoculated with seed cultures propagated in hydrolysate-free medium (**a**) or in media containing 30% (**b**), 40% (**c**) and 50% (**d**) hydrolysate. Glucose consumption (blue circle), lactic acid production (green triangle) and total CFU (black circle) are reported. Error bars indicate minimum and maximum values in duplicate experiments

When the control seed culture (i.e., unadapted cells) was inoculated into the SSF bioreactor at a cells/WIS ratio of 0.01 g_cells_/g_WIS_, no lag phase in LA production was observed at the beginning of the process and the total cell biomass was rather stable, about 10^11^ colony forming units (CFU), throughout the entire fermentation (Fig. 3a). The unadapted MA-13 converted all the glucose released from the biomass to LA in a time frame of 30 h, with average and maximum productivities of 1.11 and 1.93 g/(L h), respectively (Table 2).

**Table 2:** Effect of pre-adaptation on lactic acid production by *B. coagulans* MA 13 in SSF, using an inoculum size of 0.01 g_cells_/g_WIS_

Hydrolysate in seed medium (%)	Process time (h)	LA avg. vol. productivity (g/(L h))	LA max. vol. productivity (g/(L h))	LA avg. spec. productivity (10^−11^ g/(CFU h))	LA yield (g/g)^a^
0	30	1.11 ± 0.13	1.93 ± 0.00	0.52 ± 0.02	1.23 ± 0.05
30	15	1.74 ± 0.11	2.83 ± 0.09	1.11 ± 0.07	1.02 ± 0.12
40	24	1.23	1.91	2.06	0.97
50	24	1.19 ± 0.01	2.04 ± 0.06	1.15 ± 0.13	0.83 ± 0.04

Average ± span to minimum and maximum values

^a^g of lactic acid/g of glucose, consumed

Seed cultures propagated in media containing 30%, 40% and 50% hydrolysate were inoculated in the SSF bioreactors at the same cells/WIS ratio as was used in the control. The required process time was shortened in all the SSF experiments performed using pre-adapted seed cultures (Fig. 3 and Table 2). In particular, the time to convert all glucose to LA was shortened by half, to 15 h, for the SSF carried out using cells pre-cultured in 30% hydrolysate (Fig. 3b). Moreover, these runs showed the highest average and maximum volumetric productivities of 1.74 g/(L h) and 2.83 g/(L h), respectively, and the average specific productivity increased from 0.52·10^−11^ g/(CFU h) to 1.11·10^−11^ g/(CFU h) (Table 2). These results suggest that MA-13 was able to adapt to the inhibitory conditions of the lignocellulosic medium when hydrolysate was added to the seed cultures, which resulted in improved LA fermentation profiles in the SSF process. Therefore, the addition of hydrolysate has clear advantages over the use of hydrolysate-free seed media (Fig. 3).

Lower volumetric LA productivity and yield were achieved with the seed pre-adapted in 40% hydrolysate compared to the 30% hydrolysate pre-cultured cells. However, an additional increase in the specific LA productivity was observed (Table 2). This augmented specific productivity suggests that, at the cellular level, there is an optimum in pre-adaptation when the seed is cultivated in 40% of hydrolysate. Nonetheless, the higher concentration of biomass-derived inhibitors decreases the overall cell viability in the subsequent SSF, leading to diminished volumetric productivities. Therefore, the seed cultivation process seems to be a trade-off between good growth to achieve a large enough inoculum and a maximum pre-adaptation to provide cells that are robust enough for the subsequent SSF.

The detrimental effect of hydrolysate concentrations higher than 30% was confirmed not only by decreased average and maximum volumetric productivities but also by the longer process time (24 h) for the SSF with cells pre-cultured in 40% and 50% hydrolysate (Fig. 3c, d) (Table 2). For these reasons, seeds pre-cultured in media containing 70% and 95% hydrolysate were not further used for LA fermentations in SSF.

Altogether, these results demonstrate that the pre-adaptation of *B. coagulans* MA-13 shortens the process time and improves volumetric as well as specific LA production rates (Table 2). Therefore, MA-13 is well suited for LA production in SSF, especially in terms of total process time compared to what has been reported for other *B. coagulans* strains (Table 1).

### SSF using a (cells/water insoluble solids) ratio of 0.005 g_cells_/g_WIS_

**Fig. 4:**
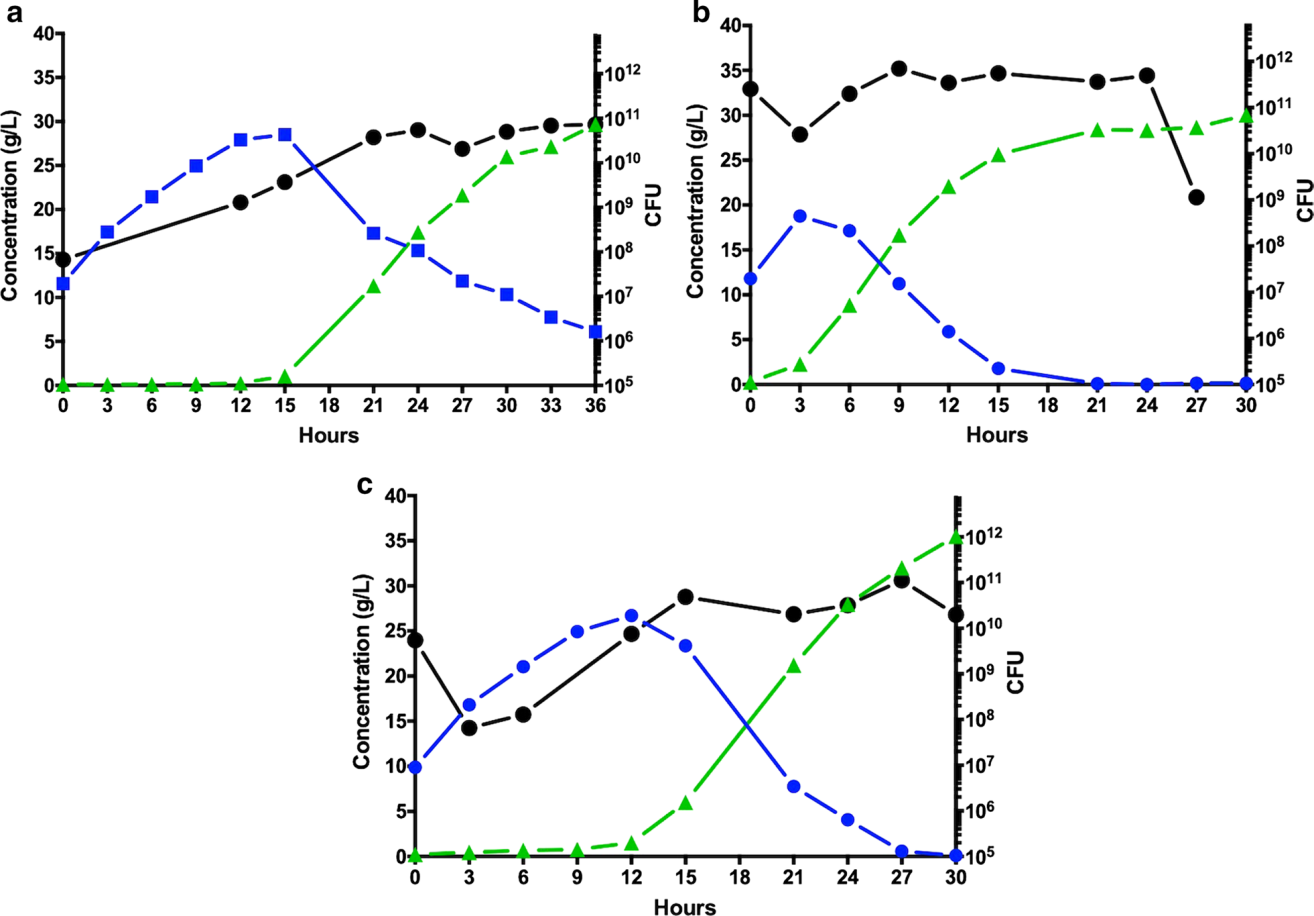
Time-course of batch SSF performed with *B. coagulans* MA-13 at a cell/biomass ratio of 0.005 g_cells_/g_WIS_. SSF experiments were carried out anaerobically in 3.6 L BioEtOH double-jacket flat-bottom vessels at 55 °C and pH 5.5. Pre-hydrolysis of the WIS fraction was carried out for 30 min by adding 10 FPU/g_WIS_ of Cellic CTec 2 before inoculating cells from the seed cultures. The fermentation medium (10% WIS from pre-treated wheat-straw) was inoculated with seeds cultivated in hydrolysate-free medium (**a**) and in media containing 30% (**b**) and 40% (**c**) hydrolysate. Glucose consumption (blue circle), lactic acid production (green triangle) and total CFU (black circle) are reported

In an attempt to test if a reduced inoculum size could still lead to efficient LA production, SSF experiments were carried out decreasing the cells/WIS ratio to 0.005 g_cells_/g_WIS_. For the unadapted control culture an initial lag phase of about 10 h was observed in the LA production (Fig. 4a, see Additional file 1 for lag phase estimates). This was likely due to an initial drop in cell viability of the unadapted culture upon transfer to the SSF slurry. In the same time frame, the total CFU increased from 10^7^ to 10^9^, reflecting an active cell growth that reached 10^11^ CFU within the first 24 h of the process (Fig. 4a). LA was produced with average and maximum volumetric productivities of 0.82 g/(L h) and 1.92 g/(L h), respectively (Table 3), whereas the total process time was considerably longer (> 36 h) than in the SSF performed with an inoculum size of 0.01 g_cells_/g_WIS_ (30 h).

**Table 3:** Effect of pre-adaptation on lactic acid production by *B. coagulans* MA 13 in SSF, using an inoculum size of 0.005 g_cells_/g_WIS_

Hydrolysate in seed medium (%)	Process time (h)	LA avg. vol. productivity (g/(L h))	LA max. vol. productivity (g/(L h))
0	> 36	0.82	1.92
30	24	1.17	2.40
40	30	1.18	2.35

In the SSF inoculated with seeds cultured in 30% hydrolysate medium, glucose was converted to LA without the occurrence of a lag phase (Fig. 4b). Thereby, the total process time was reduced to 24 h. Both average and maximum volumetric productivities increased, to 1.17 and 2.40 g/(L h), respectively (Table 3). However, in this case the process time was still 9 h longer (24 h, Table 3) than when the 30% hydrolysate pre-adapted seed was used at a ratio of 0.01 g_cells_/g_WIS_ (15 h, Table 2).

On the other hand, an initial decrease in the CFU counts and a lag phase in the lactate production when using the 40% hydrolysate-adapted seed (Fig. 4c), led to a 6 h longer total process time (30 h, Table 3) compared to the corresponding SSF experiment carried out with a ratio of 0.01 g_cells_/g_WIS_ (24 h, Table 2). This corroborates that pre-adaptation of *B. coagulans* MA-13 improves the production process, both in terms of time and productivities (Table 3). SSF performed at an inoculum size of 0.005 g_cells_/g_WIS_ were not further investigated, because the process time was, in all conditions tested, longer (Table 3) than at an inoculum size of 0.01 g_cells_/g_WIS_ (Table 2).

### Impact of pre-adaptation strategy on lactic acid production process

So far, strategies to increase the efficiency of the LA fermentation process from lignocellulose have been focused on detoxification of the solid residues by water washing (Table 1). However, this approach has the drawback of requiring large amounts of process water for the washing step, which affects both capital and operational costs by increasing the requirements for waste water treatment. These costs add to the ones related to the downstream LA separation and purification steps, which may account for as much as 30–40% of the total production costs already for pure glucose-based processes [30, 31].

Conversely, the reported pre-adaptation strategy saves costs also by decreasing the amount of clean water used in the seed culture, leading to even lower requirements for waste water treatment. Moreover, the use of a cheap carbon sources (i.e., molasses and lignocellulose hydrolysate) further decreases the cost of seed propagation. Noteworthy, this approach allows not only a cost-effective propagation of the seed culture compared to what has been reported for other *B. coagulans* strains (i.e., propagation in hydrolysate-free rich media), but it also provides a pre-adapted seed culture that performs better in SSF in terms of total fermentation time and LA productivity (Tables 2 and 3). Furthermore, a comparison of studies from the literature shows that the LA production process with the strain MA-13 in combination with pre-adaptation supersedes other reported LA production strategies with other *B. coagulans* strains in terms of volumetric productivities as well as of metabolic (g_LA_/g_glu,consumed_) and technical (g_LA_/g_glu,released_) yields (Table 1).

However, the present study does not yet give competitive results compared to pure sugar-based processes [31]. To achieve this, several additional variables would require optimization, such as the enzymatic hydrolysis, inoculum size, and total solids loading [32]. Nonetheless, the use of inexpensive fermentative substrates (e.g., lignocellulosic biomass) is presently a hot research topic, because it can allow cost-effective and sustainable large-scale production of LA by replacing the more expensive pure sugars preparations. The use of agricultural residues to produce such sugar solutions also requires substantial purification, both before and after production of the chemical [5]. However, the product purification costs in a lignocellulose-based process will likely be higher than in sugar-based, which may offset the benefits mentioned above. Therefore, the process economy can be analysed only after overall optimization of the lignocellulose-based LA production process, which is outside the scope of this study.

## Conclusions

By-products released from lignocellulosic biomass during thermo-chemical pre-treatment hamper microbial fermentation performance. The objective of this study was to test whether the pre-exposure to these inhibitors could lead to a pre-adaptation of *B. coagulans* MA-13. The reported results confirm that pre-adapted cultures show increased LA productivities. This is likely due to physiological adaptation and/or to an increased cell viability. These findings could help to pave the way for the development of a low-cost LA production process. Further development of the strain via genetic and/or metabolic strategies is envisaged to increase its robustness towards higher inhibitor concentrations.

## Methods

### Raw material

Wheat straw, pre-treated by acid-catalysed steam explosion using 1% weight/volume (w/v) H_2_SO_4_, as described in [32], was obtained from the SP Processum Biorefinery Demo Plant (Örnsköldsvik, Sweden). After pre-treatment, the biomass slurry was separated by filtration into a water-insoluble solids (WIS) fraction and a liquid fraction, hereinafter referred to as hydrolysate. The hydrolysate contained microbial growth inhibitors such as acetic acid (3.8 g/L), furfural (4.0 g/L) and HMF (1.4 g/L); for complete composition see [32]. The hydrolysate was added to the seed medium at different concentrations to obtain pre-adapted seed cultures, whereas the WIS fraction was used for LA production in SSF experiments. For the saccharification of the WIS, the commercial enzyme cocktail Cellic CTec 2 (Novozymes) was used. Enzyme activity, expressed in filter paper units (FPU), was determined according to the NREL protocol TP-510-42628 with reduced reaction volume [33].

### Strain and media

All the experiments were carried out using *B. coagulans* MA-13 [24], a thermophilic and cellulolytic strain previously isolated and cultivated using a medium containing a glycine-buffered Brock’s basal salt solution, which is suitable for the cultivation of thermoacidophilic microorganisms [34–36].

LB medium was used as inoculation medium and contained 1% (w/v) tryptone (AppliChem), 1% (w/v) NaCl (AppliChem), 0.5% (w/v) yeast extract (VWR).

The seed medium contained final concentrations of 5% (v/v) molasses, 1% (w/v) yeast extract (VWR), 1% (w/v) peptone (VWR), 0.75% (w/v) (NH_4_)_2_SO_4_ (VWR), 0.35% (w/v) KH_2_PO_4_ (VWR), 0.07% (w/v) MgSO_4_·7H_2_O (VWR), and 1× trace metals and 1× vitamins, prepared according to [37]. To test the pre-adaptation of seed cultures, hydrolysate was added at final concentrations of 30%, 40%, 50%, 70% and 95% (v/v) to the seed medium.

The SSF medium was composed of 10% weight/weight (w/w) water insoluble solids (WIS) supplemented with 1% (w/v) yeast extract (VWR), 1% (w/v) peptone (VWR) and 0.05% (w/v) (NH_4_)_2_HPO_4_ (VWR). With this setup, the approximate concentrations of the microbial growth inhibitors acetic acid, furfural and 5-hydroxymethyl furfural were 0.58 g/L, 0.61 g/L, and 0.21 g/L, respectively. All media were adjusted to pH 5.5 by titration with 3 M NaOH (Merck).

### Lactic acid production from steam-exploded wheat straw

#### Anaerobic seed pre-adaptation

All media, shake flasks and bioreactors were sterilized by autoclaving at 121 °C for 20 min before use, except the steam pre-treated wheat straw which was used without further sterilisation. Pre-cultures were started from a frozen glycerol stock and cultivated to an optical density (OD_600_) of 1.0–1.3 (about 3–4 h) in 500 mL LB medium in 2.0 L unbaffled shake flasks using a KS 4000i shaking incubator (IKA™) at 55 °C and 180 rpm. Cells were harvested by centrifugation of 100 mL pre-culture aliquots at 4000×*g* for 10 min. Pellets were resuspended in 20 mL aliquots of sterile seed medium from each bioreactor. The bioreactors were then inoculated through sterile rubber septa to an initial OD_600_ of 0.1. All seed cultivations were carried out anaerobically in 1 L of medium at 55 °C and 500 rpm in 3.6 L bioreactor vessels (INFORS HT). Nitrogen was sparged at 1 vvm. The pH was controlled at 5.5 by titration with 3 M NaOH. Antifoam 204 (Sigma-Aldrich) was added as required.

Samples were regularly withdrawn to measure OD_600_ spectrophotometrically (GENESYS 20, Thermo Scientific) as well as sucrose, lactic acid, acetic acid and acetoin via high performance liquid chromatography (HPLC). HPLC samples were centrifuged at 4000×*g* for 10 min and filtered with a 0.2 µm nylon filter before analysis. Once the seed cultures reached the early stationary phase (after 18–20 h), indicated by a decline in base titration, the cells were harvested and centrifuged at 4000×*g* for 15 min. The pellets were resuspended in 0.9% (w/v) NaCl before inoculation into the SSF bioreactors.

#### Lactic acid production in SSF configuration

Batch SSF experiments were carried out in 3.6 L BioEtOH double-jacket flat-bottom vessels (INFORS HT). Pre-hydrolysis of the WIS fraction was carried out for 30 min at an enzyme loading of 10 FPU/g_WIS_ before inoculating cells from the seed culture at a cells/WIS ratio of either 0.005 or 0.01 g_cells_/g_WIS_. After addition of cells and enzymes the initial total mass of the reactor content was 1.50 kg. Process conditions were 55 °C, 100 rpm and pH 5.5. After additional 30 min, to allow the cells to distribute evenly in the thick semi-solid SSF medium, samples were regularly collected to measure cell concentrations in the form of colony forming units (CFU) as well as glucose and fermentation products via HPLC. The fermentation process was monitored and considered to be completed when no significant changes in the lactate and glucose concentrations were detected over time. SSF experiments performed with an inoculum size of 0.01 g_cells_/g_WIS_ were carried out in duplicates. Since the production process was not improved when the inoculum size was lowered to of 0.005 g_cells_/g_WIS_, these fermentations were not further investigated and only single experiments were performed.

### Analytical procedures

#### HPLC

After centrifugation and filtration, seed and SSF samples were analysed by HPLC (UltiMate 3000, Dionex) to quantify glucose, lactic acid, acetic acid and acetoin. The analytes were identified with a refractive index and an UV detector (both Dionex) at 210 nm after separation on a Rezex ROA H+ (8%) column (Phenomenex) eluted with 5 mM H_2_SO_4_ at a flow rate of 0.8 mL/min and an oven temperature of 80 °C.

#### Cell concentration

To monitor cell growth during pre-culture and seed cultivations, OD_600_ was measured spectrophotometrically after appropriate dilution. Medium filtered through a 0.2 µm nylon filter was used as a blank. For seed cultures, cell concentrations were also measured by dry weight determination. 5.0 mL of culture medium was filtered through a pre-weighted 0.2 μm filter paper (PESU-membrane). The filter was washed three times with deionized water (MilliQ, Waters), dried at 105 °C for 24 h and weighed after temperature equilibration in a desiccator.

Culturable cell concentration was assayed by counting colony forming units (CFU). For seed cultivations, withdrawn samples were serially diluted (tenfolds) with 0.9% w/v NaCl. Given the high solids content of the SSF medium, 5.0 g of weighed samples were diluted in 0.9% w/v NaCl to a final volume of 50 mL before serial dilution. Then 100 μL of each sample was spread on LB plates in triplicate. The plates were incubated at 55 °C for 12–16 h before manual enumeration of colonies.

#### Yield and productivity calculations

The glucose content of WIS samples was measured according to the NREL protocol TP-510-42618 [38]. After two-step hydrolysis, glucose concentrations were quantified by HPLC as described above. For calculations the following assumptions were made: (i) the concentration of glucose in the liquid phase before enzymatic hydrolysis was equal to the glucose concentration in the hydrolysate fraction; (ii) all changes in the WIS occurred due to cellulose hydrolysis; and (iii) the final volume only depended on sampling and not on evaporation and base titration which were assumed to be equal (Additional file 1). Yields were calculated as lactate produced per glucose consumed [g/g] according to Eq. (1):

1 $$Y_{\text{L/G}} = \frac{{{\text{Lactate}}_{\text{produced}} }}{{{\text{Glucose}}_{\text{consumed}} }} = \frac{{m_{{{\text{Lac}},{\text{L}}\left( {t = t_{\text{end}} } \right) }} - m_{{{\text{Lac}},{\text{L}}\left( {t = 0} \right)}} + \sum m_{{{\text{Lac}},s}} }}{{\left( { m_{{{\text{Glc,WIS}}\left( {t = 0} \right)}} - m_{{{\text{Glc,WIS}}\left( {t = t_{\text{end}} } \right)}} } \right) + m_{{{\text{Glc}},{\text{L}}\left( {t = 0} \right)}} - m_{{{\text{Glc}},{\text{L}}\left( {t = t_{\text{end}} } \right)}} - \sum m_{{{\text{Glc}},s}} }},$$

where $m_{\text{Glc,WIS}}$ is the amount of glucose released during the two-step hydrolysis, $m_{\text{Glc,L}}$ is the amount of glucose in the liquid phase, $m_{\text{Lac,L}}$ is the amount of lactate in the liquid phase and $\sum m_{i,s}$ is the total amount of *i* (lactate or glucose) removed by sampling (Additional file 1). We based sampling and yield calculations on mass rather than concentration because of the particle content in the lignocellulosic slurry.

The cell-specific lactate production rate ($q_{\text{Lac}}$) was calculated according to Eq. (2). The productivity was estimated by calculating the slope of lactate concentration in the liquid phase ($c_{\text{Lac}}$, g/L) between time points *t *− 1 and *t *+ 1 corrected for the mass of the liquid fraction (mf), and dividing by the cell concentration (CFU or OD_600_) at time point *t* ($c_{X,t}$, CFU/g slurry). Assuming constant WIS content of 10% gives mf = 0.9 mL/g slurry. To account for unequal sampling intervals, the slopes between *t* − 1 and *t*, and between *t* and *t *+ 1, respectively, were weighted by the actual time intervals (Additional file 1):

2 $$q_{\text{Lac}} = \left( {\frac{{\left( {t - t_{t - 1} } \right)}}{{\left( {t_{t + 1} - t_{t - 1} } \right)}} \cdot \frac{{\left( {c_{{{\text{Lac}},t + 1}} - c_{{{\text{Lac}},t}} } \right) \cdot {\text{mf}}}}{{\left( {t_{t + 1} - t} \right)}} + \frac{{\left( {t_{t + 1} - t} \right)}}{{\left( {t_{t + 1} - t_{t - 1} } \right)}} \cdot \frac{{(c_{{{\text{Lac}},t}} - c_{{{\text{Lac}},t - 1}} ) \cdot {\text{mf}}}}{{\left( {t - t_{t - 1} } \right)}}} \right) \cdot \frac{1}{{c_{X,t} }}.$$

## Additional file

**Additional file 1.** Raw data and calculations of yield coefficients and production rates.

## Abbreviations

ATP: adenosine triphosphate
CFU: colony forming units
glu: glucose
HMF: 5-hydroxymethyl furfural
HPLC: high-performance liquid chromatography
LA: lactic acid
OD_600_: optical density
PLA: poly-lactic acid
SSF: simultaneous saccharification and fermentation
WIS: water-insoluble solids
w/v: weight/volume
w/w: weight/weight
